# Uncertainties in Steric Sea Level Change Estimation During the Satellite Altimeter Era: Concepts and Practices

**DOI:** 10.1007/s10712-016-9387-x

**Published:** 2016-10-17

**Authors:** C. R. MacIntosh, C. J. Merchant, K. von Schuckmann

**Affiliations:** 1 Department of Meteorology, University of Reading, Earley Gate, PO Box 243, Reading, RG6 6BB UK; 2 National Centre for Earth Observation, University of Reading, Reading, RG6 6BB UK; 3 Mercator Ocean, 8-10 rue Hermès, 31520 Ramonville-Saint-Agne, France

**Keywords:** In situ observations, Satellite altimetry, Uncertainty, Steric sea level, Global sea level

## Abstract

This article presents a review of current practice in estimating steric sea level change, focussed on the treatment of uncertainty. Steric sea level change is the contribution to the change in sea level arising from the dependence of density on temperature and salinity. It is a significant component of sea level rise and a reflection of changing ocean heat content. However, tracking these steric changes still remains a significant challenge for the scientific community. We review the importance of understanding the uncertainty in estimates of steric sea level change. Relevant concepts of uncertainty are discussed and illustrated with the example of observational uncertainty propagation from a single profile of temperature and salinity measurements to steric height. We summarise and discuss the recent literature on methodologies and techniques used to estimate steric sea level in the context of the treatment of uncertainty. Our conclusions are that progress in quantifying steric sea level uncertainty will benefit from: greater clarity and transparency in published discussions of uncertainty, including exploitation of international standards for quantifying and expressing uncertainty in measurement; and the development of community “recipes” for quantifying the error covariances in observations and from sparse sampling and for estimating and propagating uncertainty across spatio-temporal scales.

## Introduction

Global mean sea level (GMSL) change integrates all the volume changes of the world ocean (Church et al. 2013). Thermal expansion of sea water is a major driver of change and is highly correlated with global scale ocean heat content (OHC) (Domingues et al. 2008). Over the last 50 years, it is estimated that about 90 % of human-induced heat accumulation in the Earth’s climate system (e.g., Trenberth et al. 2014; von Schuckmann et al. 2016) has penetrated into the ocean through subduction and mixing processes, leading to an observed increase in upper OHC (Abraham et al. 2013; Church et al. 2013). The corresponding observed increase in specific volume is steric change (e.g., Levitus et al. 2005). The remaining excess heat from planetary warming goes into melting of both terrestrial and sea ice, and warming the atmosphere and land surface (Trenberth 2009; Hansen et al. 2011; Church et al. 2011; 2013). Thus, quantifying the effect of the sea water density changes on sea level variability is of crucial importance for climate change studies, as the cumulative sea level rise can be regarded as an important climate change indicator, as well as being of direct societal importance.

The dominant component of steric sea level change is temperature-related, i.e. the thermosteric component. Salinity variations associated with freshwater tendencies at the sea surface and redistributed in the ocean’s interior have a negligible effect on sea water density and thus on sea level changes on the global scale (e.g., Lowe and Gregory 2006). On regional to basin scales, the role of halosteric effects through the addition and subtraction of freshwater or mixing processes can be large and should not be neglected in sea level studies (e.g., Durack et al. 2014; Gille 2004). Regional freshwater changes are found to have an important imprint on global mean sea level (Boening et al. 2012), but their relation to global halosteric sea level changes has not yet been quantified.

Three approaches to evaluate steric sea level from observations are available. The first is direct estimation from the global ocean in situ observing system, from data available back to the 1950s (e.g., Levitus et al. 2005, 2012). Observations have been mostly limited to the upper ocean (700 m) before the year 2005 due to data sampling issues (Abraham et al. 2013). From 2005 onwards, data sampling has strongly increased (Roemmich et al., 2009), and improved global scale estimates of steric sea level down to 2000 m are now possible (e.g., von Schuckmann et al. 2009). Hydrographic observations from sparse and irregular in situ sampling of the deep ocean exist and show that deep ocean layers (>2000 m) contribute around 0.1 mm per year to global steric sea level change (Purkey and Johnson 2010). Also analyses based on the Coupled Model Intercomparison Project Phase 5 (CMIP5) model simulations highlight the fundamental role of deeper ocean layer temperature changes (below 2000 m depth) to global ocean warming (Palmer and McNeall 2014; Cheng et al. 2016) and thermosteric sea level increase (Lorbacher et al. 2014).

A second method to obtain steric sea level estimates is to use results from ocean reanalyses, which are the combination of ocean models, atmospheric forcing fluxes and ocean observations via data assimilation methods. Ocean reanalyses can, in principle, provide more accurate information than observation-only- or model-only-based ocean estimations (Trenberth et al. 2014). However, methodological uncertainty, deficiencies in the observing system and model biases are major obstacles for the reliable reconstruction of the past ocean climate (Balmaseda et al. 2015; von Schuckmann et al. 2016). Intercomparisons of ocean reanalyses deliver insights into the performance of data assimilation systems, the underlying physical models and adequacy of the ocean observing system, and results suggest that upper layer (<700 m) global thermosteric sea level from ocean reanalyses is comparatively well constrained by observations, in contrast to large uncertainty in the deep ocean (>700 m) and halosteric contributions (Storto et al. 2015). These results emphasise the need to better observe the deep ocean, both for providing observational constraints for future ocean state estimation efforts and also to develop improved models and data assimilation methods (Palmer et al. 2015).

The third method is based on global sea level budget studies by using remote sensing data (e.g., von Schuckmann et al. 2014). Full depth global steric sea level change can be derived from the difference of total sea level change from satellite altimetry (Cazenave and Llovel 2010) and the change from ocean mass change measured using gravimetry (Chambers et al. 2010). Results based on this method underpin the significant role of deep ocean warming (Rietbroek et al. 2016), but uncertainties in the different observing systems are too large to quantify the contribution below 2000 m depth (von Schuckmann et al. 2014). Indirect steric estimates through the sea level budget are still restricted to the period from the year 2002 onwards where satellite gravimetry data are available. However, this indirect method is ideal to monitor the quality of global observing systems in the context of physical budget constraints.

This paper is a review of direct estimation of the steric component of global sea level (i.e. of the first of the three methods described) paying particular attention to questions of uncertainty. It is written in the context of a Special Issue of the journal addressing related topics about sea level budget. First, we introduce some basic concepts of steric sea level in Sect. 2. The main focus of this review is how such differences can be accounted for in terms of the inherent uncertainties present in constructing datasets representing steric changes in global sea level. Section 3 presents the theoretical principles applicable to constructing within a dataset rigorous estimates of those uncertainties, highlighting the practical challenges. In Sect. 4, practices of dataset construction and uncertainty estimation that have been reported in the literature are reviewed, illustrating the variety of approaches. In the light of these practices, some conclusions are drawn in the final section.

## Basic Concepts

### Calculating Steric Sea level

The integration of the hydrostatic balance equation gives the vertical depth (“thickness”) of water between two pressure levels, *p*_1_ and *p*_2_:

1 $$Z = - \mathop \smallint \limits_{{p_{1} }}^{{p_{2} }} \frac{1}{g\rho }dp$$

Density [*ρ*] is a function of temperature* T*, salinity* S* and pressure* p*. To find a form for calculating steric effects on the thickness, *Z*, between these pressures, a common approach is to recast this expression for a profile (*T*, *S*) using a first-order expansion of the specific volume, $\theta$ (inverse of density) around a reference profile (*T*_0_,* S*_0_), which can be either a standard sea water reference or, commonly, a climatological background field.

2 $$Z = - \frac{1}{g}\mathop \smallint \limits_{{p_{1} }}^{{p_{2} }} \left( {\theta_{0} \left( {T_{0} ,S_{0} , p} \right) + \frac{\partial \theta }{\partial T}\left( {T - T_{0} } \right) + \frac{\partial \theta }{\partial S}\left( {S - S_{0} } \right)} \right)dp$$

**Fig. 1:**
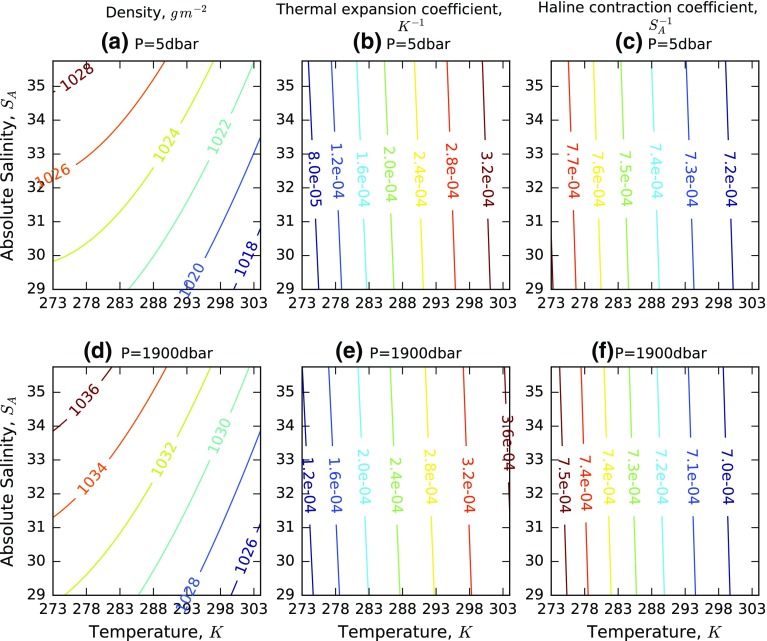
Dependence of density (**a**, **d**) on temperature and salinity at two pressures (*upper*, 5 db; *lower*, 1900 db). *Centre*: thermal expansion (**b**, **e**, *α*, K^−1^) and right: haline contraction (**c**, **f**, −β, g^−1^ kg) coefficients, which derive from the partial derivatives of the density surface

Defining the “steric thickness”, *h*, as the difference from the thickness of the reference profile (i.e. *h* = *Z* − Z_0_) and transforming the vertical integration into depth coordinates* z*, the steric thickness can be expressed in terms of the thermal expansion coefficient ($\alpha = \frac{1}{\theta }\frac{\partial \theta }{\partial T})$ and haline expansion coefficient ($\beta = \frac{1}{\theta }\frac{\partial \theta }{\partial S}$). The total steric effect separates cleanly into a thermosteric component, $h_{T}$, and a halosteric component, $h_{S}$:

3 $$h = h_{T} + h_{S} = \mathop \smallint \limits_{z1}^{z2} \alpha \left( {T - T_{0} } \right)dz + \mathop \smallint \limits_{z1}^{z2} \beta \left( {S - S_{0} } \right)dz$$

(adapted from Antonov et al. 2002). The *α* and *β* coefficients quantify the fractional change in density (or, equivalently, in specific volume) per unit increase in temperature and salinity, respectively. A temperature increase causes an increase in specific volume (expansion). A salinity increase decreases specific volume; hence, the positive values shown in Fig. 1 are coefficients of contraction (negative expansion).

### Equation of State

Steric sea level change is not fully correlated with OHC change in that the former reflects the ability of water to expand and contract, rather than its heat capacity; a given heat uptake can produce different steric height changes depending on the initial conditions. Density of sea water is a function of temperature and salinity at any given pressure and is described through the Thermodynamic Equation of Seawater, referred to as “TEOS-10”. (Pawlowicz et al. 2012, IOC, SCOR and IAPSO 2010). TEOS-10 is the SI-traceable standard for relating temperature, salinity and density relationships in sea water in the limit to the thermodynamic properties of other substances (especially ice and humid air) and replaces the previous standard, EOS-80 (UNESCO 1981a, b).

TEOS-10 uses absolute salinity,* S*_A_ (units: g kg^−1^), which is the mass fraction of dissolved salts in a parcel of water. This replaces the previous standard practical salinity unit, psu, which is simply a measure of the conductivity of the water. Thus, absolute salinity cannot be directly measured and is related to the conductivity (which is measured) via a reference standard composition in the TEOS-10 equations. Absolute salinity is defined as *S*_R_ = 35.16504 g kg^−1^ (from North Atlantic surface waters) for the standard reference composition sea water (Millero et al. 2008). Natural sea water contains many other substances in somewhat variable proportions, e.g., dissolved silicates. Where there is known to be systematic deviation from the standard relationship between conductivity and* S*_A_, empirical corrections to salinity are used (particularly in the Pacific). These correction factors are outlined in McDougall et al., (2012). As a single measurement of salinity must always be a simplification of the true composition of sea water, the measurement, representation and even the definition of salinity continue to evolve (e.g., Wright et al. 2011).

The dependence of density on temperature and salinity shows that the thermal expansion coefficient *α* (units: K^−1^) depends on both salinity and pressure, with effects of up to ~3 % and ~30 % across the particular range (Fig. 1a, d). However, the most influential factor on *α* is temperature itself, which causes a variation of a factor of 3 (Fig. 1b). Likewise, the haline contraction coefficient −*β* (units of g^−1^ kg) is dependent on salinity itself and pressure, but it most strongly dependent on temperature (Fig. 1c, f). This means, for example, that for the most accurate estimation of thermosteric effects, knowledge of the salinity profile as well as the temperature profile is required.

**Fig. 2:**
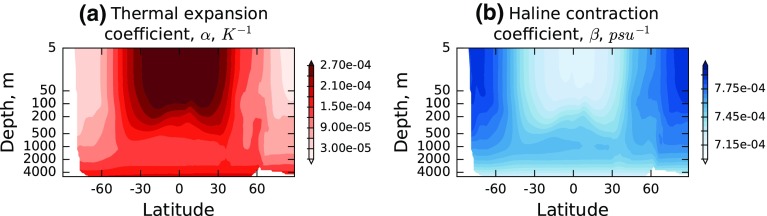
Zonal average of *α* (units: K^−1^) and −*β* (units of g^−1^ kg) as a function of depth, from EN4 (Good et al. 2013)

Given these dependencies, the climatological variation of thermal expansion and haline contraction coefficients can be examined, as in Fig. 2. An increase in ocean temperature in the tropical ocean at depths down to ~500 m is seen to be much more effective in causing sea level rise than the same increase in the colder waters poleward of 60° of latitude or at greater depths. This is mainly determined by the temperature-dependence of *α*, and the variation of *β* has a similar spatial shape because its variation is also temperature-determined; however, the dependence has the opposite sign, with least sensitivity of specific volume to salinity in the upper tropical ocean, as expected from Fig. 1c.

## Uncertainty in Steric Sea Level Change

One can readily find different usages for the terms “error” and “uncertainty” in the scientific literature, but these terms have unambiguous, internationally agreed definitions (JCGM 2008), adherence to which brings conceptual precision. “Error” is the difference between the true value and the measured value of a quantity; since the true value is unknown and unknowable, errors in measured values must also be unknowable. “Uncertainty” is the degree of doubt about a measured value: given the result of a measurement or calculation, it quantifies within what dispersion of values around that result is it reasonable to assume that the (unknown) true value lies (JCGM 2008). Uncertainty is usually quantified as the standard deviation of the estimated probability distribution of error in the measured value.

### Importance of Uncertainty

Estimates from multiple groups of global mean steric sea level may show substantial spread in terms of the observed trend and its uncertainty (e.g., Table 2). However, the discrepancies can give an indication of the level of uncertainty. The magnitude of disagreement, and of uncertainty, increases with increasing spatio-temporal resolution, in general. It is important also to attempt to estimate uncertainty within an analysis (“internal estimates”) and not only to look at the disagreement between different results (“external analysis”). If internal and external estimates of uncertainty are inconsistent, it is a sign that the origins of errors in the analyses are not adequately understood. For example, agreement between groups of estimates can be unrealistically good (external uncertainty estimates are biased small), if all or many groups adopt approaches that lead to a degree of commonality of errors across analyses. This can be revealed if rigorous internal uncertainty estimates suggest that uncertainty is much greater than disagreement across the group.

This section of the paper discusses principles of “internally” estimating uncertainty in steric sea level variations. The aim is to outline what is involved in developing a comprehensive estimate. This is intended as background information for any readers who may be only partly aware of methods of uncertainty estimation, to inform the discussion of practices found in the literature in Sect. 4.

### Key Uncertainty Concepts

Fully to understand what we can and cannot infer about GMSL rise, calculations of the steric contribution need to be associated with estimates of their uncertainty.

For complex datasets, uncertainty estimation is generally challenging since it requires significant effort to gather fundamental uncertainty information and significant computation to correctly propagate and combine uncertainties to give a final estimate. Faced with this challenge, the temptation is to make simplifying assumptions (such as assuming uncorrelated errors) and/or address only those sources of error that are reasonably well understood. As a result, uncertainty is more likely to be underestimated than overestimated.

The overall uncertainty in a climate dataset can be decomposed into structural uncertainty and value uncertainty (Thorne et al. 2005). In constructing a climate dataset, many choices have to be made. In the case of an analysis of steric sea level change, choices include: which sources of *T*–*S* profile data to include; what quality control is applied to profiles; what bias corrections to apply to profiles; which auxiliary data to use, such as a reference climatology; what method of data gridding and/or interpolation to apply; what method of vertical integration to use; and what choice of density state function to assume? The dispersion of outcomes that could arise for all well-justified choices reflects the structural uncertainty. Thorne et al. (2005) noted that dataset producers traditionally do not estimate structural uncertainty at all.

Value uncertainty is the total remaining uncertainty given the structural choice made for constructing the dataset. It includes the uncertainty due to sampling, i.e. the facts that the available measurements are finite and not ideally (or even adequately) distributed. Uncertainty in parameters used in calculations (e.g., **α**) contributes to value uncertainty. Most obviously, the uncertainty in individual measured values of temperature, salinity and pressure propagates to uncertainty in the steric sea level and their estimate is challenging in practice.

Decisions regarding the construction of a dataset may influence both structural and value uncertainty. For example, the uncertainty associated with forming a gridded product from limited sampling as individual profiles has a structural component, which depends on the choice of method (kriging vs. simple binning, and the method used to fill gaps), and a value component, which could in principle be formally propagated (for example, uncertainty associated with random and systematic measurement errors in the profile, and associated with the estimated unsampled variability).

Any calculation of steric SL involves the combination of many measured values, typically profiles of *T*, *S* and* p*. A key question is therefore how uncertainty in these variables propagates to give the steric SL uncertainty. It is not only the magnitude of uncertainty in each measured value that affects the combined uncertainty: any correlation between errors in measured values greatly affects the combined uncertainty, as will be discussed in the next section.

### Uncertainty of Steric Sea Level Estimate from a Single Profile

Using the formulation of steric SL of Eq. (3) as a starting point, we consider what would be involved in a detailed estimation of the uncertainty in $h_{T}$, which is the thermosteric component, from a single profile of *T* against depth. The same principles would apply to the halosteric component and the *S* profile.

First, note that in formulating Eq. (3), we have already made choices that could be made differently, and which contribute to structural uncertainty. These include: the means of evaluating the expansion coefficient at each level; and the numerical integration scheme used, which makes implicit assumptions about the variation of the integrand between the levels at which measured values are available.

Second, we consider estimating the value uncertainty. In general, the numerical implementation of the integral can be written (exactly or approximately) as a linear combination of measured values at *N* measurement levels:

4 $$f = \mathop \sum \limits_{n = 1}^{N} w_{n} \alpha_{n} \left( {T_{n} - T_{0n} } \right) + 0$$

where $w_{n}$ expresses the weight of the nth set of measurements in the profile gets in the integrated result, which depends on the separation between levels and the nature of the numerical scheme used. The relevance of the “+ 0” term will be made clear below. Now, let all the parameter and measured values contributing to f be collected in a column vector **x**, for example:

5 $$\textbf{x}^{\text{T}} = \left[ {\begin{array}{*{20}c} {\begin{array}{*{20}c} {\begin{array}{*{20}c} {T_{1} } & \cdots & {T_{N} } \\ \end{array} } & {\begin{array}{*{20}c} {\begin{array}{*{20}c} {S_{1} } & \cdots & {S_{N} } \\ \end{array} } & {z_{1} } & \cdots \\ \end{array} } & {z_{N} } \\ \end{array} } & {\begin{array}{*{20}c} {T_{01} } & \cdots & {T_{0N} } \\ \end{array} } & {\begin{array}{*{20}c} {S_{01} } & \cdots & {S_{0N} } \\ \end{array} } \\ \end{array} } \right]$$

The ordering within **x** is arbitrary. It contains all measured values and parameters—including the reference profiles (which may affect $\alpha_{n}$) and the depth estimates (which affect $w_{n}$).

A full evaluation of the uncertainty, under a first-order approximation, is given by

6 $$u\left( {h_{T} } \right) = \sqrt {{\mathbf{c}}^{{\mathbf{T}}} {\mathbf{Uc}}} + u\left( 0 \right)$$

where

7 $${\mathbf{c}} = \frac{\partial f}{{\partial {\mathbf{x}}}}$$

which is a vector of sensitivity coefficients, corresponding term-by-term to the contents of **x**. This vector can be evaluated straightforwardly by differentiation. So, for example,

8 $$c_{1} = \frac{\partial f}{{\partial T_{1} }} = w_{1} \left( {T_{1} - T_{01} } \right)\frac{{\partial \alpha_{1} }}{{\partial T_{1} }} + w_{1} \alpha_{1} \cong w_{1} \alpha_{1}$$

assuming the thermal expansion coefficient is evaluated using only the reference profile and is insensitive, therefore, to $T_{1}$.

The matrix **U** is the error covariance matrix for the elements in **x**. This has to be developed from an understanding of the measurements and parameters. The element of U for row *i* and column *j* is

9 $$U_{ij} = u\left( {x_{i} } \right)u\left( {x_{j} } \right)r\left( {x_{i} ,x_{j} } \right)$$

where $u\left( {x_{i} } \right)$ is the magnitude of uncertainty in value $x_{i}$ and $r\left( {x_{i} ,x_{j} } \right)$ is the correlation coefficient between errors in $x_{i}$ and $x_{j}$. Note that it is errors (not uncertainties) that can be correlated. This happens, for example, when a common effect (source of error) contributes to the total errors in both $x_{i}$ and $x_{j}$.

Estimating **U** therefore involves developing an understanding of the magnitude of the uncertainty in every measured value and parameter, and having a model for the degree of correlation between errors in different elements of **x**.

The 0 terms in the definitions of *f* and the $u\left( 0 \right)$ terms remind us that the total uncertainty, $u\left( {h_{T} } \right)$, is more than the propagation of the value uncertainties. Discrete data have been vertically integrated using a numerical scheme, which itself is a source of numerical uncertainty. A well-chosen integration scheme will be unbiased, hence the “+ 0” formulation, and will provide an estimate of the numerical uncertainty, $u\left( 0 \right)$.

The above principles are very general and well established. To make them concrete, consider a simplified case of a profile of temperature measurements obtained by a particular sensor. This temperature sensor records digitised output, which effectively acts as a source of noise in individual measurements. The sensor is calibrated to a stated accuracy, and the calibration error is independent of temperature. Let us assume that these are the only error effects. (This scenario is just for illustration: a real sensor’s error structure would likely be rather more complex.)

If the temperature data are quantised in bins of width *t*, the standard uncertainty that this introduces is

10 $$u_{\text{digit}} \left( {T_{i} } \right) = \frac{t}{2\sqrt 3 }$$

from considering the standard deviation of a top-hat distribution of full width *t*. Where vertical gradients in temperature are very gradual relative to *t* and the vertical sampling interval, there could be some correlation in the error in measured values, but in general for this sensor we assume that this is not the case and treat the digitisation error as random and independent between measured temperatures. So,

11 $$r_{\text{digit}} \left( {T_{i} ,T_{j} } \right) = \delta_{ij}$$

where $\delta_{ij}$ = 0 when* i* ≠ *j* and is 1 when* i* = *j*.

In contrast, the calibration error here is a constant bias. This is an example of a systematic effect, which means an effect that could be corrected for in principle if better information were available (a broad definition that includes the case of constant bias). Here, if calibration were performed again more precisely before deployment, a correction could be estimated. But all corrections are imperfect, and a smaller systematic error would remain, after this correction, associated with a smaller residual uncertainty.

Estimating the magnitude of uncertainty for an error effect can be done variously by statistical means (e.g., repeated laboratory evaluations), by simulation of the measurement process, by sourcing relevant information from published literature, by physical reasoning, etc. In this case, the uncertainty from calibration might be estimated from the manufacturer’s stated goal for the calibration accuracy, corresponding to an uncertainty, $u_{calib} \left( {T_{i} } \right) = u_{t}$. Given the assumed nature of the error (constant bias), $r_{\text{digit}} \left( {T_{i} ,T_{j} } \right) = 1$ and so

12 $$U_{ij} = \frac{{t^{2} }}{12}\delta_{ij} + u_{t}^{2}$$

In a more realistic case, there may be several error-causing effects to combine with more complex correlation structures. Modelling the uncertainty contribution of all significant effects to **U** is a significant effort, but a full error covariance model would need to be developed only once for each source of profile data. While **U** is profile-specific (for example, the depths of measurement levels are different for each profile, and therefore, vertical correlation coefficients may differ), developing a community “recipe” for the error covariance matrix is feasible. Several studies address measurement error covariances (e.g., Kaplan et al. 2000; Levitus et al. 2012, hereafter L12, Gaillard et al. 2016), although in practice diagonal error matrices are usually implemented to reduce the substantial computational cost. Neglecting the correlation terms (off-diagonal terms in the matrix) results in an underestimation of steric thickness uncertainty that is potentially large.

Since the error in measured values is a priori independent of errors in parameters, the same recipe for the observation error covariance matrix would apply to a variety of structural/methodological choices, since the full covariance matrix can be constructed of a “data” block and a “parameter” block:

13 $${\mathbf{U}} = \left[ {\begin{array}{*{20}c} {{\mathbf{U}}_{{{\mathbf{data}}}} } & 0 \\ 0 & {{\mathbf{U}}_{{{\mathbf{param}}}} } \\ \end{array} } \right]$$

This subsection has presented the general equations for uncertainty estimation in a calculated quantity given the input measured and parameter values. Their application has been illustrated by a simplified case of calculating the uncertainty in the thermosteric influence on the thickness of a layer of sea water arising from the *T* profile uncertainties. Similar steps would address the uncertainty from the *S* and *z* values, as well as from the assumed parameters. The concepts behind the uncertainty analysis are well established, but systematic estimation of the necessary parameters, describing the dispersion and correlation of errors from the major effects, requires a significant level of effort.

### Propagating Uncertainty Through Gridding, Interpolation and Integration

To create a full in situ-based estimate of the steric contribution to GMSL from a dataset of profiles involves gridding and/or interpolation, and spatio-temporal integration. One approach is to aggregate the steric thickness estimates from profiles onto a spatio-temporal grid, propagating the uncertainty results for individual thickness estimates to the gridded product. Alternatively, *T* and *S* observations may be gridded/interpolated, and steric sea level found by integrating Eq. 3 for those fields, the most commonly used method in the literature (Sect. 4). Gridded datasets aggregate available estimates for selected layers within the ocean on a spatio-temporal grid, typically 1 deg in latitude/longitude and monthly, or coarser. In many cells, there will be no or few data, the proportion being greater the less coarse the spatio-temporal grid. Nonetheless, aggregation of more than one set of observations will be necessary in some cells. The aggregation of two or more observations should account for their relative uncertainty, for example, by weighting more uncertainty data less heavily, and this is straightforward if uncertainty estimates have been associated with each observation. In addition, the limited sampling within the grid implies that, even for cells where data are available, there is a statistical uncertainty from having subsampled the natural variability over the bounds of the cell.

In principle, a full uncertainty model for gridded, interpolated or integrated SL estimates can be built using the principles and equations presented for a single profile in the previous subsection.

The same principles and equations for uncertainty propagation discussed in the previous section also apply to these transformations. Each of the transformations used in averaging to a grid, interpolating to give a complete field and integrating over ocean volumes can be viewed as scaling and reweighting the influence of individual observations in the final result(s). Each transformation can be recast in a form similar to Eq. (4), and Eqs. (6) and (7) can be used to propagate uncertainty to the final result, at least in principle. For gridded/interpolated/integrated products, the number of individual variables rapidly becomes large. The difficulties of propagating observation uncertainty are therefore to associate uncertainty and error correlation information with each observation and deal with the practicalities of organising the computation efficiently.

Two additional considerations arise: the modelling of uncertainty from sparse sampling and the use of “background fields” in interpolation.

The sampling uncertainty, when gridding observations, is an example where “u(0)” can be very significant. The number of vertical profiles at a grid location is usually small, and a given profile gives only a snapshot of the subgrid variability within that column. Thus, sampling uncertainty depends on the magnitude and nature of variability within the space–time box defined by the grid and the time–space pattern of sampling in the observational data. The observations themselves may have limited ability to provide an estimate of the variability, and additional information from ocean models and reanalyses may be useful in constructing a model for variability, such as magnitude of variations in *T* and *S*, the correlation length scales of variability in time and space and the degree of covariability between *T* and *S*, e.g., Cheng and Zhu (2016). The uncertainty from different patterns of observational sampling can then be estimated via simulation (Monte Carlo) methods.

Background fields may be used in interpolation. Interpolation procedures for sparse observations rely on the geophysically justified assumption that variability is correlated in space and time, so that the observations are informative about variability beyond the time and location of observation. Where no observations are sufficiently close to be informative, interpolation methods generally rely on a background field, such as climatology, to provide the most highly weighted estimate. In such areas, the interpolation uncertainty is larger, tending towards the uncertainty of the background field—e.g., the estimate of climatological variability. In terms of uncertainty estimation, the background field may be treated the same way as actual observations, albeit that the uncertainty will be relatively large. Note that use of a static climatology as a background field can cause bias (underestimation of change) in the face of a real geophysical trend, as has been discussed in reference to GMSL and OHC (e.g., Lyman and Johnson 2008; Boyer et al. 2016).

**Fig. 3:**
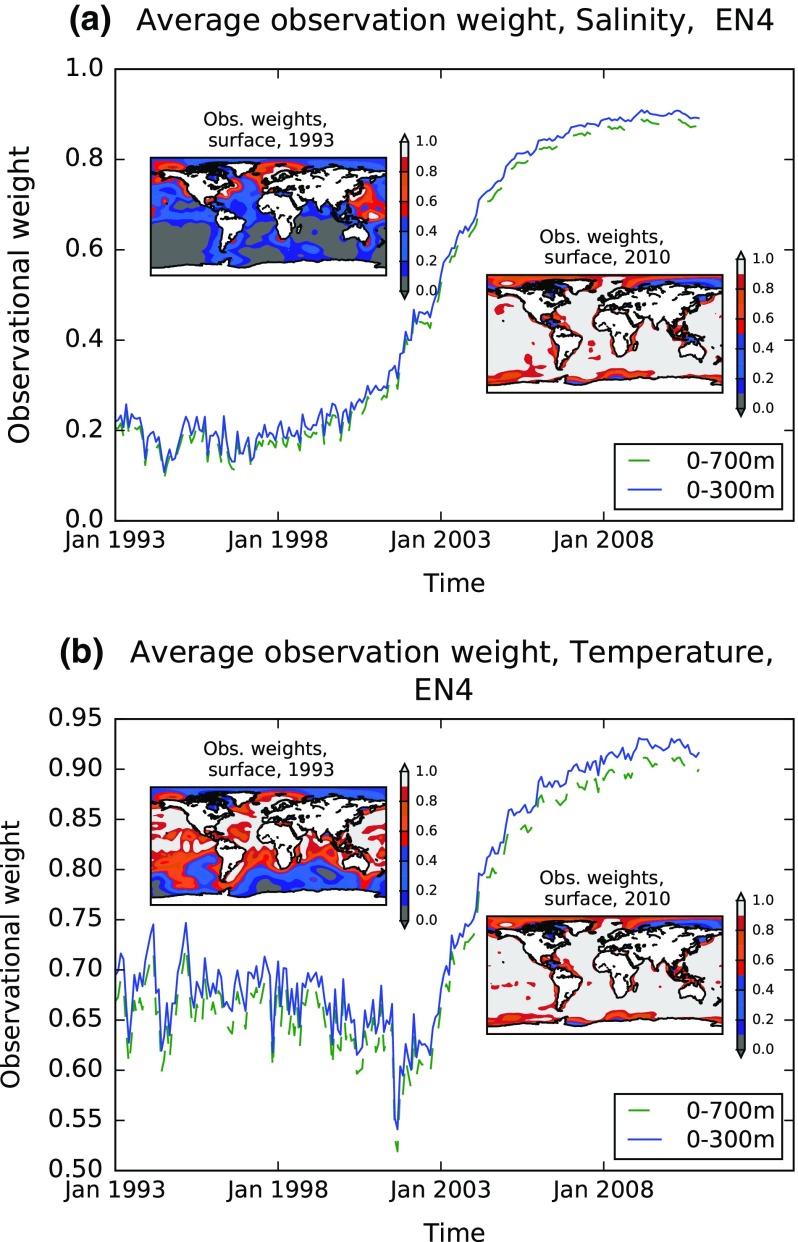
Global mean observational weights from EN4, for the 0–300 m (*blue*) and 0–700 m (*green*, *dashed*) layers, salinity (**a**) and temperature (**b**). *Inset panels* illustrate the spatial variability of observational weights at the surface at the beginning (*top left*) and end (*lower right*) of the time period

The weight of observations in determining the estimate for a particular analysis cell can be output from the interpolation method. Where the weight tends to zero, the interpolated value reflects only the background estimate. The fundamental limitation of data in estimating GMSL from in situ data alone is illustrated in Fig. 3. This figure relates to the EN4 dataset (Good et al. 2013) and shows the weight of profile observations for the surface layer of the interpolated analysis, which is an interpolation at 1 × 1 degree resolution in latitude and longitude and is monthly in time. The observation weight has been averaged globally for two depth ranges (see legend), and thus, 1 minus this weight indicates the overall dependence on the background field. Since the ocean volume gaining observational weight from the presence of one or more profiles in the grid box depends on vertical, horizontal and temporal correlation length scale parameters, it is principally the relative change in observation weight over time and variation in space that is instructive here. (With different estimates of the length scale parameters, the observation weights would have a different value, but would show similar spatial and temporal changes.) The steep rise in global mean observation weight over time between the year 2001 and 2005 principally shows how effective the programme of deployment of Argo profiling floats has been at improving the sampling of ocean profiles.

Several studies have discussed the impact of sparse sampling on trends (e.g., Cheng et al. 2015; Abraham et al. 2013). On average, the choice of the mapping method for irregularly sampled in situ measurements is the largest source of uncertainty (Boyer et al. 2016). Despite the tremendous technical developments of the ocean in situ observing system through the implementation of the Argo array, coverage is not yet truly global. The deep ocean below 2000 m (nearly half the volume) has very few measurements. The few that are available are from sparse, but very precise, hydrographic sections from research vessels (L12; Desbruyères et al. 2014). There are also gaps in the geographic coverage, with almost no floats in marginal seas (such as the Indonesian Sea; von Schuckmann et al. 2014), under sea ice or polewards of 60° (von Schuckmann et al. 2016). As a consequence, steric sea level estimates still differ at subseasonal to interannual timescale (Trenberth et al. 2016; Dieng et al. 2015; Abraham et al. 2013; VS11) and even show significant disagreement at decadal scale (von Schuckmann et al. 2016).

Another recent analysis (Good 2016) has analysed quantitatively the impact that sparse sampling can have on the reconstructed temperature trends over the global ocean. The study subsamples a spatially complete model ocean to construct a pseudo-observational dataset and then compares global mean temperature estimates from this pseudo-observational set with the model “truth”. The study concludes that there is substantial scope for background climatology to poorly represent the true temperature fields (see also Lyman and Johnson 2014) and for this influence of this climatology to cause systematic errors in reconstructed time series. The study also found that poor spatial coverage in the pre-Argo era also had the potential to introduce spurious variability comparable in magnitude to the true variability. The modelling of sampling uncertainty is thus seen to be critical to fairly estimating the uncertainty in steric sea level (see also Boyer et al. 2016).

A fundamental challenge is the sparseness of observations below 2 km, throughout the period to the present. Uncertainty about deep ocean (>2 km depth) heat content change and associated steric effects is profound. For example, Purkey and Johnson (2010) provide only basin-scale estimates of SL change below 2000 m, as sampling density is insufficient to resolve trends at higher spatial resolution. Their estimate of the uncertainty on the global mean trend is of similar order to the trend itself (0.113 ± 0.100 mm year^−1^) where the uncertainty estimate is a measure of the 2σ variability in the temperature trend in each region, area weighted, converted to SL coordinates and combined in quadrature. Thus, although plausibly a modest component of steric sea level rise (seemingly between 0 and 20 % of the total), the uncertainty in this deep ocean change is a non-negligible component of the overall trend uncertainty.

## Data and Uncertainty Methods in the Literature

### Global Mean Estimates

While the focus of this review is the estimate of steric SL change from in situ measurements, here we discuss briefly other methods of estimation. Estimates of SL change that are truly independent can confirm or refute each other and also be used as diagnostic tools to highlight areas where our understanding may be limited.

**Fig. 4:**
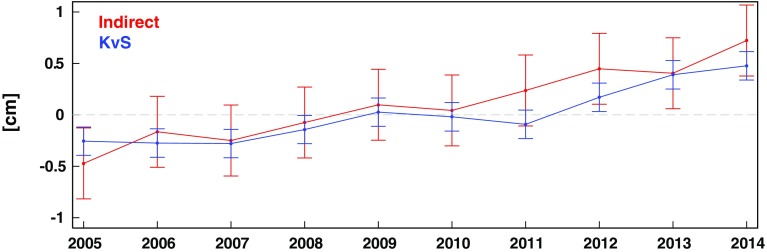
Annual mean estimates of global thermosteric sea level during 2005–2014 from the surface down to 2000 m depth based on Argo measurements (updated after von Schuckmann and Le Traon 2011 (KvS, *blue*), and the indirect estimate through the sea level budget (*red*). Method, data use and uncertainty estimates for the KvS time series are described in von Schuckmann and Le Traon (2011). For the indirect approach, the evaluation method and the use of GRACE data are described in von Schuckmann et al. 2014; for estimates of total sea level, the gridded product from ESA CCI is used (product version V1.1_20151113, see http://www.esa-sealevel-cci.org/products and Ablain et al. 2015 for more details)

Statistically robust upper ocean warming directly related to steric rise was found in both current in situ and indirect estimates (Fig. 4), but both of these approaches nonetheless face important challenges or limitations. The indirect estimate is bounded by data system availability, starting in the year 2002 (beginning of GRACE time series), and associated uncertainties are still too large for the extraction of warming trends given by Purkey and Johnson (2010) of about 0.1 ± 0.1 mm year^−1^ below 2000 m depth (von Schuckmann et al. 2014, Fig. 4). For the direct approach, uncertainties in OHC estimates arise from calculating global fields from temporally and spatially irregular data (mapping method), instrument bias corrections and the definitions of a baseline climatology from which anomalies are calculated (Boyer et al. 2016; Lyman and Johnson 2014; Abraham et al. 2013).

Fundamental advances in observing systems (e.g., Argo, gravimetry), in reanalysis systems (Balmaseda et al. 2015), in in situ data bias correction methods (Boyer et al. 2016) and in estimates of deep ocean contributions from in situ measurements (e.g., Purkey and Johnson 2010) have led to significant improvements in global steric sea level estimates over time. The warming of the upper 700 m during 1970–2014 (1993–2014) caused an estimated mean thermosteric rate of rise of 0.8 ± 0.2 mm year^−1^ (0.9 ± 0.2 mm year^−1^) (90 % confidence), which is 30–40 % of the observed rate of GMSL rise (Cheng et al., 2015, Chambers et al. 2016). Steric contributions in the 700–2000 m depth layer account for 0.2 ± 0.1 mm year^−1^ over the altimeter era and about 0.1 ± 0.1 mm year^−1^ below 2000 m depth (Purkey and Johnson 2010, Chambers et al. 2016). Decadal steric trends for the Argo era (2005–2014) are of similar size and amount to 0.8 ± 0.2 mm year^−1^ for the upper 2000 m depth (Table 1; Fig. 3) and 0.9 ± 0.2 mm year^−1^ for the entire ocean depth when taking into account the Purkey and Johnson (2010) estimate. In summary, the most recent steric trend estimates based on the direct and indirect approach are in considerable agreement with associated error bars (Fig. 4).

**Table 1:** Most recent estimates from the GRACE/Argo “golden” era starting in 2005 (note that Argo programme has started in the year 2000, but has reached near global coverage from the year 2005 onwards, e.g., von Schuckmann et al., 2009). Trends are quoted as the mean plus or minus the reported uncertainty based on the spread of results for different estimates of a given method

Method	Steric sea level trend (mm year^−1^, 2005–2014)
Direct estimate	0.8 ± 0.2 (0–2000 m) 0.9 ± 0.2 (full depth, this study (Fig. 3) and Purkey and Johnson (2010))
Indirect estimate	1.2 ± 0.4

 The indirect estimate as derived from the sea level budget is an updated version of the von Schuckmann et al. (2014) methodology, except that the ESA CCI dataset is used (see figure caption of Fig. 3 for more information on the data used), taking into account error bars of both satellite altimetry (Ablain et al. 2015) and ocean mass from GRACE (see von Schuckmann et al. 2014 for more details on error estimates). A review on data processing and uncertainty methods in the literature is further discussed in Sect. 4.2 and 4.3.

### Temperature and Salinity In Situ Measurements

In constructing a SL analysis and uncertainty budget, investigators will generally undertake quality control and correct for known problems with the data. Biases in the temperature measurement systems have been extensively studied and can be both widespread and systematic in nature (e.g., Abraham et al. 2013, Cheng et al. 2015). As discussed in Sect. 3, there remains post-correction uncertainty that ideally should be estimated. More generally, the selection, quality control and bias correction of input data all contribute to differences SL analyses, reflecting structural uncertainty (e.g., Cheng et al., 2015). A basic choice is whether to build the analysis from raw profile data, or a dataset that has already undergone some processing to remove unreliable profiles and/or correct for biases. Table 3 summarises the input data, additional data sources and interpolation method for several studies.

The in situ observing system is a complex and constantly evolving network of instruments. Substantive discussion of the system requires careful and complex analysis and is outside the scope of this study; however, here we highlight two issues that are areas of substantial ongoing research. The first is the sparse and constantly evolving nature of the in situ observing system. This presents substantial challenges to any potential analyst. Spatially and temporally sparse sampling is not spatially uniform, and temporal coverage (together with quality of coverage) can vary substantially by region. The construction of a spatially and temporally uniform representation of the Earth’s oceans from such input data is addressed in many studies (e.g., Good 2016; Ishii and Kimoto 2009; von Schuckmann and Le Traon (2011) and can be the major source of structural uncertainty in estimates of thermosteric SL change (Boyer et al. 2016).

Second, the evolving nature of the observing system presents its own challenges. Each observing solution has its own strengths, limitations and biases, which often evolve in time, or with instrument design changes. Particularly in the pre-Argo era, systematic removal of fall speed biases has been a major focus of quality control (QC) efforts. The biases may depend on more than one factor, for example, water temperature variations (Abraham et al. 2016) or XBT (eXpendable Bathythermograph) or MBT (Mechanical Bathythermograph) model (Abraham et al. 2013). Several current correction schemes exist and are in widespread use, for example Ishii and Kimoto (2009), Levitus et al. (2012), Wijffels et al. (2008) and Gouretski and Reseghetti (2010), and the selection of a given method contributes to differences between products as structural uncertainty (Boyer et al. 2016). Abraham et al. (2013) present a substantial review of bias correction methods and results for non-Argo type in situ observing instruments, and a comprehensive review of the current state of knowledge of these systems.

A detailed analysis of the in situ observing system, its strengths and limitation and its evolution over time is outside of the scope of this work; however, a recent major review article by Abraham et al. (2013) addresses all of these issues comprehensively.

Any search of the literature will reveal a large number of estimates of steric SL change from in situ data. Table 2 summarises recent estimates of SL change from analysis of temperature and salinity in situ measurements, from Domingues et al. 2008 (the basis for the estimates used in IPCC AR5,^1^ Church et al. 2013; note the “short names” in the table which will be used for brevity hereafter), to present day. It does not include estimates from reanalyses, which have a more complex relationship between input data and SL estimate, or indirect estimates from satellite data, which act as independent data that should ideally be explained in terms of the in situ estimates (Table 1).

**Table 2:** Summary of recent literature estimates of global mean sea level trend

Study	Short name	Trend, mm year^−1^	Uncertainty estimate mm year^−1^	Depth(m)	Time	Spatial resolution (degrees)	Temporal resolution
Ishii and Kimoto 2009	IK09	0.294	±0.057	0–700	1951–2005	1 × 1	Monthly
Levitus et al. 2012	L12	0.41	–	0–700	1955–2010	1 × 1	Annual
Levitus et al. 2012	L12	0.54	–	0–2000	1955–2010	1 × 1	Annual
Llovel et al. 2013 (L12)	LL09	0.42	±0.12	0–700	1960–2010	1 × 1	Quarterly
Llovel et al. 2013 (IK09)	IK09	0.39	±0.12	0–700	1960–2010	1 × 1	Monthly
IPCC, Church et al. 2013	AR5	0.6	±0.3	0–700	1961–2003	1 × 1	Monthly
Domingues et al. 2008	D08	0.52	±0.08	0–700	1961–2003	1 × 1	Monthly
Storto et al. 2015 Objective analyses	StOA	1.11	±0.08	Full depth	1993–2010	1 × 1	Monthly
Ishii and Kimoto 2009	IK09	1.23	±0.295	0–700	1993–2005	1 × 1	Monthly
Cabanes et al. 2013	CORA	0.64	±0.12	10–1500	2005–2010	5^°^ Lat, 10^°^ Lon	Monthly
Cabanes et al. 2013	CORA, Argo only	0.58	±0.1	10–1500	2005–2010	5^°^ Lat, 10^°^ Lon	Monthly
Von Schuckmann and Le Traon 2011	VS14	0.5	±0.1	10–1500	2005–2012	5^°^ Lat, 10^°^ Lon	Quarterly

Most of the estimates are not directly comparable as they cover different time periods and depths. This illustrates a persistent problem in reviewing the literature and assessing the consensus on sea level trends. This highlights the need for more systematic, coordinated efforts. Currently, it is difficult and time consuming to compare methodological differences or improvements as time and depth considerations can strongly affect trends over these short (on ocean circulation timescales) time periods. Nonetheless, on comparing estimates with similar or identical analysis domains, it does appear that methodological differences result in substantially different trends.

The estimates in Table 2 are not independent of each other in terms of source data: common or overlapping source datasets are used, inevitably. Most of the estimates that include the pre-Argo period use their contemporary version of the World Ocean Database (IQuOD) also addresses (Boyer et al. 2013), sometimes supplemented by other data sources. WOD aims to provide the most comprehensive available temperature and salinity profile data and undergoes a continuous programme of update and review. Those estimates which cover only the Argo period, and use information only from Argo floats, may get their information direct from the Argo teams (e.g., VS14). However, data from the Argo floats are also included in the WOD data store; therefore, none of the estimates is wholly independent of others in terms of source data (Table 3).

**Table 3:** Construction of SL trend estimates from temperature and salinity in situ profiles**—**summary of methods from the literature

	Domingues et al. 2008	Levitus et al. 2012	Ishii and Kimoto 2009	Von Schuckmann and Le Traon 2011	EN3 (Ingleby and Huddleston 2007)	CORA (Cabanes et al. 2013)	ARMOR (Guinehut et al. 2012)
Short name	D08	L12	IK09	VS14	EN3	CORA	ARMOR
Input data	EN3	WOD 2009	WOD 2005	Argo only	WOD 2005	Coriolis	SST, altimeter, EN3, Coriolis
Additional data	No	Yes, see text	Yes, see text	No	Yes, see text	No	No
XBT bias correction	Wijffels et al. 2008	L12	IK09	N/A	Wijffels et al. 2008	Hamon et al. 2012	N/A
MBT	No	Yes	Yes	N/A	Yes	No	N/A
Argo bias correction	Yes their own QC	Yes Argo teams’ corrections	Yes Ignore biased data	Yes Argo teams’ corrections	No	Yes Argo teams’ corrections	Yes Argo teams’ corrections
Background climatology	Alory et al. 2007	Locarnini et al. 2010 (WOA)	Locarnini et al. 2010 (WOA)	Von Schuckmann et al. 2009	WOA 98	Not stated	N/A
OI/binning method	Reduced space optimal interpolation	Objective mapping	Objective mapping	Binning, box averaging	Bell et al. 2000	VS11	N/A
Sparse data treatment	Kaplan et al. 2000	Relax to climatology	Relax to climatology	Gap filled from climatology	Relax to climatology	VS11	N/A
Time-varying salinity	No	Yes	No	Yes	Yes	Yes	Yes

 Two global data centres exist, i.e. NODC (https://www.nodc.noaa.gov) and Coriolis (http://www.coriolis.eu.org). However, each study may also make use of some auxiliary datasets and conduct additional QC on their input database. IK09 use XBT observations from the Global Temperature and Salinity Profile Programme (GTSPP), Japanese Maritime Self-Defence Force (JMSDF) and sea surface temperature data (Ishii et al. 2006). L12 use data to extend the World Ocean Atlas (WOA) from 2009 to the end of 2010. EN3, which forms the input to two of the studies (D08 and Storto et al. 2015), comprises WOD05 plus data from GTSPP, Argo and the Arctic Synoptic Basin Wide Oceanography (ASBO) project (Ingleby and Huddleston 2007), CORA comprises data from the Coriolis data centre, comprising European ship observations, XBT and other profiling systems, and Argo data. The data are global from 1990 onwards. ARMOR use all *T* and *S* profiles from the EN3 dataset, with the exception of those labelled as Argo. Argo data are from the Coriolis data centre up to 2009 and combine these data with SST and satellite altimeter data.

Additional quality assurance (QA) procedures may be used to retain only the “best” data, or, as in IK09 and EN3, to thin the data where many profiles exist in a single location. Thinning the data substantially reduces the cost of optimal interpolation (OI) in the Ishii et al. (2006) data set, allowing several estimates of trends using different QA procedures to be evaluated.

These five distinct starting datasets are used to construct seven gridded products. (D08, L12, IK09, VS11, EN3, CORA and ARMOR) and, from these, twelve estimates of steric SL trends (Table 2). Of the studies reviewed here, IK09 and D08 do not include salinity and therefore strictly provide an estimate of thermosteric SL rise. The other estimates do include time-varying salinity.

### Published Sea Level Trends

During the construction of their estimates of SL change, researchers must make decisions at each step regarding data and methods. Many decisions reflect reasoned judgements, where alternative conclusions could also be defended. This is precisely the value of multiple groups addressing themselves to such work: the different approaches adopted ensure there is some exploration of structural uncertainty across different attempts. The degree to which the structural uncertainty is explored is difficult to assess where no systematic intercomparison of methods has been attempted. There are also procedures that may suppress some structural uncertainty: results of new methods are inevitably compared during development with published estimates, which may lead to reduced diversity of outcome. Nonetheless, a useful step in understanding the degree to which structural uncertainty is explored is reviewing the range of data and method choices present in the current literature. This section is a contribution to such a review and summarises the nature of several key choices about data and method. These are presented against the background of the uncertainty concepts introduced in Sect. 3; we discuss, to the degree possible from a literature review, how each operation might contribute to a formal uncertainty budget and how uncertainty is handled at each stage of the process.

There is substantial community effort to address these apparent discrepancies, by systematically comparing different methods for construction (e.g., Llovel et al. 2013), although these often focus on ocean heat content (e.g., Lyman and Johnson 2014; Cheng et al. 2016; Boyer et al. 2016). A particular community effort had been developed to quantify uncertainties in OHC estimates which arise from calculating global fields from temporally and spatially irregular data (mapping method), instrument bias corrections and the definitions of a baseline climatology from which anomalies are calculated (Boyer et al. 2016). In this context, the International Quality controlled Ocean Database also addresses this issue through a systematic quality analysis of the historical record aiming to define internationally agreed standards and guidance.

### Spatial Structures of Steric Sea Level Change

The global mean estimates of steric SL change are associated with substantial regional variability. This is important when discussing interpolation and data manipulation as sparse sampling in some locations may have a disproportionately large effect, for example, if the water is warm or salinity effects are important. These regional effects are illustrated using data from the EN4 analysis (Good et al. 2013).

**Fig. 5:**
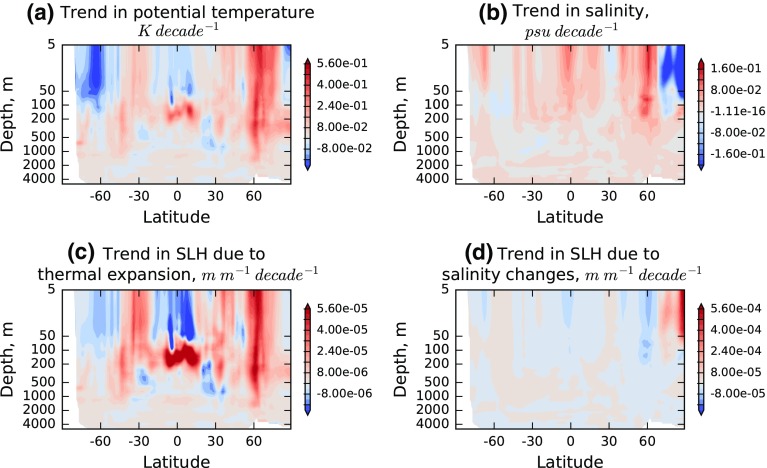
Temperature and salinity trends for 1993 to 2010 (**a**, **b**) and the resulting trend in thermosteric and halosteric sea level change (**c**, **d**), expressed in metres of sea level rise per metre depth of ocean, per decade

Strong warming trends can be observed at northern mid-latitudes to a depth of several hundred metres (Fig. 5) and are known to be located in the North Atlantic (Rhein et al. 2013). These are accompanied at very high latitudes by freshening trends (Fig. 5b), although salinity sampling in this region is sparse, in particular before the Argo era, so the relative strength of this feature may be poorly constrained (Good et al. 2013).

**Fig. 6:**
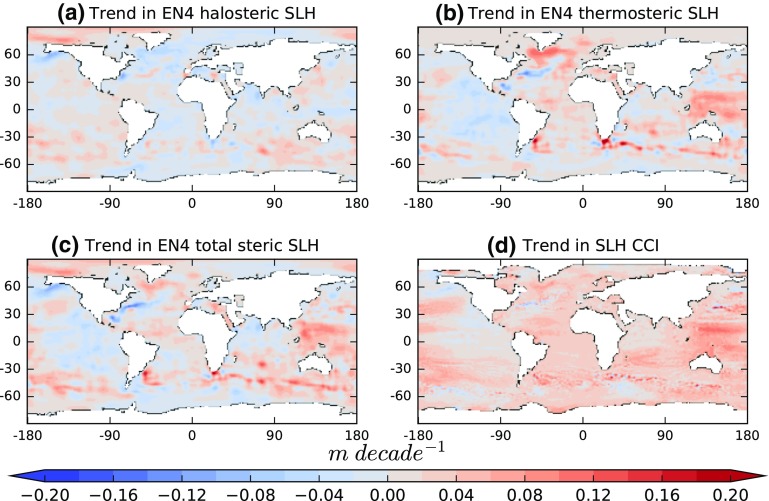
Spatial distribution of **a** halosteric trend, **b** thermosteric trend, **c** halo- and thermosteric (total steric) trend and **d** total sea level trend (SLH) from satellite altimetry from the ESA CCI project. All trends are evaluated over the period 1993–2010

The monthly, gridded data are converted to contributions to sea level (SL) change relative to the time mean via the expansion and contraction coefficients (Eq. (3), Fig. 2). Both α and *β* are, primarily, functions of temperature (rather than salinity) given pressure (Fig. 1), so trends in temperature and salinity have the most effect in the tropics and at depths of the order of a few hundred metres or less. The larger relative impact of the subsurface tropical warming on SL change is very clear in Fig. 5c. Note that the halosteric trends (Fig. 5d) for this period have a smaller effect on regional SL than do the observed temperature trends; however, there are some areas where they are not negligible, for example around 60 °N. The total trend in SL is calculated by integrating the gridded product vertically (Fig. 6a–c). For comparison, the total observed SL trend, from ESA’s Climate Change Initiative (CCI) Sea Level project (Ablain et al. 2015), was shown (Fig. 6d). The regional impact of salinity in the North Atlantic reduces the total steric trend substantially relative to the thermosteric trend alone, in common with previous studies (e.g., Durack et al. 2014)

Overall, mean estimates of global mean steric sea level from different methods have reasonable consistency, although there is considerable spread in individual estimates. Such spread reflects uncertainties in data and methods that are not fully understood and quantified. The global mean reflects the net effect of trends that have considerable spatio-temporal structure and, thus, good understanding of uncertainty in key areas such as the North Atlantic and Pacific oceans is required.

### Interpolation and Regridding/Rebinning

Interpolation and gridding sparse and generally inhomogeneous data are a source of structural and sampling uncertainty, additional to the uncertainty from propagation of value uncertainties. This section discusses briefly the methods used to produce gridded products.

Interpolation methods can generally estimate the statistical uncertainty (which may be referred to as “error” estimate) in interpolated values, based on some underlying model of spatio-temporal error covariance in the input data. Value uncertainty estimates are often used in interpolation to influence the relative weights of observations and the background field, for example. This doesn’t in itself ensure full propagation of value uncertainty through the interpolation procedure, if value error correlation structures are simplified or neglected. Uncertainty from limited sampling may be accounted for in the interpolation process using statistics of variability that are estimated to account for unsampled variability on scales of more than one grid cell; sampling uncertainty associated with the relationship between individual profile measurements and grid cell means (so called “intra-box” effects, see Kaplan et al. 2000) should be explicitly recognised (e.g., as in Gaillard et al. 2016).

L12 conduct an objective analysis as described in Locarnini et al. (2010) to produce a gridded dataset. IK09 also use an objective analysis technique, from Derber and Rosati (1989), as do EN3 (described in Bell et al. 2000).

These techniques must all use some estimate of time and length scales across which profiles may be correlated, in order to weight the observations. EN3 use a simple assumption that length scales are invariant across the globe and use values of 300 km latitude and 400 km longitude. The method used in the L12 study comprises several iterations of their objective analysis method using different length scales of influence, as a method of capturing different scales of variability, from 888 to 444 km (Locarnini et al. 2010). IK09 use spatially varying decorrelation scales of 300 km in the horizontal and 10 m in the vertical at the sea surface, and which increase linearly by 30 km/100 m and 6 m/100 m away from the surface.

Neither L12 nor EN3 use an explicit temporal correlation window, although the EN3 for any given month are constructed using the previous month’s estimate as input, so some correlation is implicitly included. The decorrelation timescale in Ishii et al. (2006) is 15 days at the sea surface and increases linearly as 2 days/100 m.

D08 use an optimal interpolation technique described in Kaplan et al. (2000), which explicitly calculates a space covariance matrix over the analysis time period. This method includes instrumental error in the analysis and also attempts to account for intra-box sampling uncertainty by calculating the box variance, under the assumption that the error [sic] in the box value is related to high-frequency variability within the box.

ARMOR also use an optimal interpolation method, but merge in situ data with satellite products of SST and altimetry. This study optimally interpolates temperature and salinity fields which have been synthesised from satellite data, with the *T* and *S* in situ measurements. The synthetic fields are generated using statistical relationships between surface and subsurface measurements, compiled using in situ data from the well-sampled Argo period.

CORA and VS14 use the method of von Schuckmann and Le Traon (2011, hereafter VS11). Both studies use a box averaging method rather than interpolation. Within each 5 degree latitude × 10 degree longitude × 3 month box, profiles are averaged using a weighted average. The weights are represented by a covariance matrix between each pair of observations, where the values are calculated using correlation scales between pairs of observations of 15 days and 150 km. This addresses the variable sampling density across the dataset by ensuring that more comprehensively sampled regions are not over-represented. For reported uncertainties, VS11 use the method of Bretherton (1976) to weight the grid box variance by the inverse of the covariance matrix. The resulting error is, if the correlation terms are non zero, larger than an estimate in which each observation is assumed to be independent.

### Considerations Regarding Background Climatology

The majority of the studies construct a background climatology to be used in dataset construction and also commonly for gap-filling sparse data. The construction and choice of the ocean climatology used for interpolation requires care (Gaillard et al. 2016). For example, the rapidly increasing density of observations over the past two decades means that a simple average over the available observations would be biased, and the extent of the bias would be spatially varying, as some areas of the globe have been proportionately better sampled for longer. Various methods are used to construct climatologies to avoid this and similar pitfalls.

L12 and IK09 use annual climatologies from the WOA (Locarnini et al. 2010). The climatology is the average of five 10-year climatologies, which gives a reweighting that approximately accounts for the much greater volume of data in later years. In addition, a constraint of vertical stability has been imposed on the temperature/salinity climatologies—i.e. potential density of one level is nowhere lower than the density of the next shallowest level. Unstable climatological profiles can arise because there are many more temperature than salinity measurements and because of errors in measured values. The climatologies are often used to fill data gaps in regions where observations are sparse. L12 and previous estimates by Levitus et al. deal with sparse data by constructing running 5-year estimates of sea level at 1 × 1 degree horizontal resolution. Temperature anomalies are constructed for each 1 degree mean temperature value by subtracting the WOD climatology (Locarnini et al. 2010). Despite these anomalies being composited over a running 5-year period, some gaps in the record still remain. This method uses the WOD background climatology described above to fill gaps and assumes zero anomaly where there is no direct information.

EN3 combine the WOD 98 climatology with their objective analysis from the previous month. The resulting analysis therefore contains some signal persistence through the record. The EN3 background field also relaxes to climatology in the absence of data profiles, reducing the weight of the observations relative to the climatology gradually over 300–400 km. This background field is then used as input to the objective analysis for the following month, effectively widening the temporal window over which observations can have an influence.

Some studies construct their own climatology from the input data. D08 use an optimal interpolation technique developed by Ridgway et al. (2002), which accounts for variable sampling densities at different locations via a weighting function, which also takes into account local bathymetry and land barriers.

VS11 use reference climatology from a previous study (von Schuckmann et al. 2009) to fill gaps in individual Argo profiles. As this study deals only with the Argo era, changes in sampling density need not be a consideration. However, the effect of the choice of the background climatology has been taken into account in the error bar of the global VS11 estimate.

Both IK09 and VS11 make some investigation of the effect of the choice of background climatology. VS11 use a second climatology and calculate the standard deviation of the difference between the two resulting global mean time series. The final estimate of uncertainty is calculated as the quadratic sum of the global mean, area-weighted uncertainty and the uncertainty associated with the climatological time series.

IK09 compare the use of the WOA climatology for interpolation with using their own climatology, as the long-term time mean from the XBTs after they have applied their correction is quite different (0.236 ± 0.066 and 0.294 ± 0.077 mm year^−1^—see their Table 5 for the full comparison of their estimates exposing structural uncertainty).

### Uncertainty in Trends and Global Means

Uncertainty in a geophysical trend comprises two elements: the statistical uncertainty in fitting a trend line to a finite length of time series in the face of geophysical variability and errors in measured points, plus the uncertainty in the stability of the observing system. Stability is the constancy of the systematic error between the measured values and the truth. The latter component is often neglected, not so much because it is known to be negligible, but because stability is difficult to assess. In the case of steric sea level, the changes in the observing system described earlier do raise the likelihood that systematic effects in the observing system have changed over time. The most obvious instability is the switch from dominance of XBT to Argo profiles, given that different corrections to which these different sources are subject. Moreover, global sampling density had significantly decreased during this transition period of changing observing systems (e.g., Cabanes et al. 2013). To estimate the observational stability, the temporal correlations of errors across the time series could be quantified, although in practice this is complex. This is an area where further research will be beneficial.

It is often unclear exactly what, in terms of uncertainty, is accounted for in quoted uncertainty bounds. Estimates of uncertainty in the published literature may not be directly comparable as they are not representing the same uncertainties. This can lead to apparent contradictions within or between studies, unless the precise nature of the bounds is explicitly made clear.

In some cases, quoted uncertainties in trends are based upon the statistical uncertainty. Here, the question arises as to whether auto-correlation along the time series is properly accounted for: not doing so will tend to underestimate trend uncertainty.

This highlights the need to enhance the discussions of uncertainties for global estimates and to find a common way to directly compare the different approaches and their related scientific interpretations.

In this review, L12, IK09, VS11, LL12, D08 and CORA propagate in some way the uncertainties of the gridded product to the final, global mean estimate. Each study propagates uncertainty into its final estimate differently, in addition to the range of methods used to incorporate uncertainty information discussed above and the types of uncertainty that are accounted for in each estimate.

For example, L12, using the method described in Antonov et al. (2002), combine standard errors from the gridded product as a weighted sum (where the weights are the partial derivatives with respect to the dimension being combined, or as standard RMS if the errors are considered independent) to give global mean error estimates. However, the uncertainties quoted for the trends reflect the statistical uncertainty in the straight line fit and do not exploit any of the previously calculated uncertainty information explicitly. The evolution of the observing system over time raises the likelihood of observational instability (temporal covariance of errors) in steric sea level time series, as well as decreasing uncertainty for the recent decade. It is relatively common for uncertainty estimates in the global mean to be used to provide weights when fitting a trend line. VS11 and CORA use a weighted least squares fit for their trend uncertainty estimation, in which the weights are the propagated global mean uncertainty weights as discussed in Sect. 5. LL09 use the Levitus et al. (2009) gridded temperature uncertainties and weight them according to vertical and horizontal correlation length scales. As in VS11, these weights are used to calculate weighted least squares fit for the final quoted trend. D08 also propagate gridded errors into the global mean using their chosen interpolation method (Kaplan et al. 2000), although it is unclear whether these estimates are propagated into the fitted trends in the final estimate. Generalised least squares (GLS) methods (Aitken 1934) yield trend estimates that account for estimated data uncertainty and temporal error covariance. Although more complex to implement, application of GLS may reveal trend uncertainty to be greater than previously found using (weighted) ordinary least squares.

Iterative approaches to trend uncertainty also are used. Storto et al. (2015) do not provide information on grid box level or time series uncertainty. Rather, they provide as uncertainty estimates the 95 % confidence limits on their trend using a bootstrap (i.e. subsampling of variability) method. Intercomparison of a range of trend estimation methods would increase understanding of their applicability to the case of steric sea level change.

## Discussion

The present study reviews estimates of steric SL with a particular focus on uncertainty estimates. Resolving the problem of representing global mean steric SL change using point measurements, which provide a subsample of the full ocean state, requires considerable effort and scientific rigour. Many scientifically sound methodological choices are possible. Within the current literature, the diversity of approach is substantial (e.g., compare VS11, L12, D08) and provides a useful method of exposing structural uncertainty. Diversity of approach is strength. Nonetheless, in any area of science, structural uncertainty can be underestimated. Thus, there is a need for realistic “internal” assessment of uncertainty, as well as by looking at the diversity of results.

Sampling uncertainty is a major contributor to total uncertainty and surely is dominant for certain epochs and ocean domains. Nonetheless, value uncertainty should be assessed via rigorous uncertainty propagation and accounting for the correlated nature of errors, not least as a contribution to constraining the steric change in parts of the ocean that are inadequately observed. Community efforts are underway with the Global Ocean Data Assimilation Experiment (GODAE) Ocean View (https://www.godae.org/OSSE-OSE-home.html) and the European initiative AtlantOS (https://www.atlantos-h2020.eu/) through observing system evaluations and observing system simulation experiments (e.g., Halliwell et al. 2014). However, these efforts should, in future, be supported by a community effort to quantify error covariance estimates for classes of profile observation, in addition to the extensive existing body of work on observational bias (e.g., Abraham et al. 2013)

Historically, there has been enormous community effort to maintain, improve and understand the in situ record (e.g., Wijffels et al. 2008; Hamon et al. 2012; Abraham et al. 2013; Boyer et al. 2016 and references therein). Large-scale corrections to the in situ record can have a substantial impact on the evolution of estimated SL change (e.g., D08, IK09). As the true state of the ocean can never be known, it is important that not only is the impact of the corrections assessed (IK09), but that inevitable post-correction uncertainties are also acknowledged and, where possible, estimated.

Recently, as recommended under the CLIVAR^2^ research focus CONCEPT-HEAT (http://www.clivar.org/research-foci/heat-budget), a systematic comparison of different methods on common data is encouraged. For the pre-Argo era, such an activity has started under the project IQuOD (www.iquod.org), and a community paper is under way (Boyer et al. 2016). Still, efforts are needed for Argo era data—this, and the underlying international collaboration are of particular importance to support observing system’s development into the future.

The development of the Argo observing network has undoubtedly been the single largest modifier to our understanding of the global oceans in recent years. There is of course the need of continued international maintenance, and, if possible extend (e.g., to the deep ocean), the in situ observing system; but there is also scope to improve our exploitation of existing data; through the systematic comparisons outlined above, through formal methodological assessment and through comparison with independent data such as that from the ESA CCI projects (Ablain et al. 2015; Merchant et al. 2015)

Other systematic approaches can include the use of modelled “test” data, to compare methods and aspects of methods by testing their ability to reproduce “known” model results under realistic sampling (Good 2016). Such approaches should be expanded into systematic benchmarking studies (Chandler et al. 2012) to build maximum trust in steric sea level products.

In the absence of such systematic benchmarking comparisons, it can be difficult to assess whether observed differences in SL estimates and their associated uncertainty estimates are consistent. While some individual studies do discuss some aspects of structural uncertainty (e.g., VS11, Lyman and Johnson 2014, IK09), systematic comparison across studies is not generally facilitated and this may mask important differences that arise from changes in the state of the global oceans. The time period under consideration can, for example, have a substantial effect not on the trend estimate, but also on the impact of methodological choices and bias corrections. In the case of XBT bias corrections, the effect in D08 and IK09 is, on the face of it, very different (0.262 ± 0.063 to 0.236 ± 0.066 mm year^−1^ in IK09, an increase of “about 50 %” on previous literature estimates in D08). Closer examination of the time periods under discussion, however, reveals that similar features in the global mean time series are resolved in each case when the bias correction is applied, and that the extra 10 years of ocean data in IK09 alter the slope of the trend, but not the shape of the features in the common time period. A true assessment of this and of any similar case is limited; however, as the D08 study does not explicitly compare the same method, merely referring to previous estimates in the literature, and methodological differences cannot be ruled out.

These approaches also require the deployment of clear (unambiguous) and concise language for the discussion of error and uncertainty. In the absence of such a language, even when studies are comparing like time periods, it may not be obvious that different studies mean very different things by their quoted uncertainty estimates. Commonly, but not exclusively, the quoted uncertainty in a trend is provided using a standard least squares fit. However, the fit may (or may not) be weighted by uncertainties propagated from an optimal interpolation method (VS11), may be estimated by a bootstrap subsampling method (Storto et al. 2015) or may simply be a measure of the deviation of the fit from a straight line (L12). Wherever a quoted uncertainty estimate in a trend is based only on the fitting uncertainty in the face of geophysical variability, important aspects of the true trend uncertainty, such as the degree of instability of the observing system, are omitted. Thus, even if the meaning of each quoted uncertainty is clear, different meanings of quoted uncertainties make interpretation and comparisons between studies difficult (e.g., Table 1). It cannot necessarily be assumed that the result with the smallest quoted uncertainty is in reality the most certain trend estimate.

Developing a well-characterised uncertainty estimate is intrinsically beneficial. Clear discussion of uncertainty sources and formal propagation of uncertainties allow better understanding of differences between datasets, improving our understanding of the underlying physical quantities. Systematic assessment of whether differences in estimates are consistent with their quoted uncertainties is a useful diagnostic of the degree of real understanding of the phenomenon. Results that agree too well (within quoted internal uncertainties) can indicate some lack of independence (“herding” effects) in the development of the datasets, raising concern about the true uncertainty of a consensus picture. In a field in which most studies share at least some input data, clear discussion of uncertainty must be necessary to tease out such effects and their sources. Where results diverge more than expected given their quoted uncertainties, this suggests there is significant influence on the result of factors whose implications are not yet well understood (at the level of the community working in the area).

Historically, ocean scientists have pioneered international approaches to global science. Large-scale data collection and data quality initiatives are often internationally coordinated. Significant progress may accrue from coordination for the development of improved measurement uncertainty information, innovative, rigorous systems of uncertainty propagation and systematic approaches to intercomparison, such as benchmarking. Efforts to establish error covariance estimates and uncertainty estimates for both the current observing network, and, where possible the historic record, are already underway (e.g., Guinehut et al. 2012, CLIVAR, IQuOD). Likewise, progress can be made via further coordinated development and comparison of methods of estimating “intra-box” sampling uncertainty (e.g., Kaplan et al. 2000; Ingleby and Huddleston 2007; Good et al. 2013) and large-scale sampling uncertainty, via coordinated experiments in the reconstruction of known model fields (e.g., Good 2016).

There is therefore great scope to develop community discussions around formalising approaches to uncertainty. Progress in quantifying steric sea level uncertainty will benefit from greater clarity and transparency in discussions of uncertainty in the scientific literature. Rigorous, shared understanding across the community of how uncertainty in measurements should be expressed will enable researchers to better understand and build on the results of others and improve comparability of uncertainty estimates. Progress may be accelerated by developing and sharing rigorous community “recipes” for common problems relating to correct quantification of uncertainty. We identify in this review the potential value of such recipes for quantifying the error covariances in observations and from sparse sampling and for estimating and propagating uncertainty across spatio-temporal scales. International standards for the estimation, propagation and expression of measurement uncertainty exist which are applicable to the problem of steric sea level change. The overall conclusion of this review is to emphasise the importance of progress in quantifying and expressing uncertainty with greater rigour.

## Footnotes

1 The Fifth Assessment Report (AR5) of the United Nations Intergovernmental Panel on Climate Change (IPCC).

2 CLImate and ocean: VARiability, predictability and change. This is one of the four core projects of the World Climate Research Programme (WCRP).
